# Patient-reported Outcomes of Transperineal Reanastomosis for Recurrent or Obliterative Post-prostatectomy Vesicourethral Anastomotic Stenosis

**DOI:** 10.1590/S1677-5538.IBJU.2026.0112

**Published:** 2026-04-20

**Authors:** Jakob Klemm, Katharina Oberneder, Navid Roessler, Robert J. Schulz, Max C. Wagner, Victor M. Schuettfort, Tim A. Ludwig, Margit Fisch, Roland Dahlem, Malte W. Vetterlein

**Affiliations:** 1 University Medical Center Hamburg-Eppendorf Department of Urology Hamburg Germany Department of Urology, University Medical Center Hamburg-Eppendorf, Hamburg, Germany; 2 Medical University of Vienna Comprehensive Cancer Center Department of Urology Vienna Austria Department of Urology, Comprehensive Cancer Center, Medical University of Vienna, Vienna, Austria

**Keywords:** Anastomosis, Surgical, Patient Reported Outcome Measures, Urinary Sphincter, Artificial

## Abstract

**Purpose:**

Post-prostatectomy vesicourethral anastomotic stenosis (VUAS) affects up to 5% of patients. A subset of cases remains refractory to endoscopic treatment, significantly impacting quality of life in the long-term. This study presents 15-year follow-up data on patients undergoing open reanastomosis for refractory or obliterative VUAS.

**Patients and Methods:**

We included patients who underwent transperineal vesicourethral reanastomosis from 2009–2023. The procedure was often staged, with artificial urinary sphincter (AUS) implantation three months later. Co-primary endpoints included retreatment-free survival (RFS) and patient-reported outcome measures (PROMs). RFS was analyzed with Kaplan-Meier estimators, and PROMs were evaluated per scoring manuals. Additionally, we conducted a scoping review of all studies reporting outcomes following complex VUAS reconstruction.

**Results:**

Among 46 patients with VUAS, prior endoscopic interventions included dilation (17%), incision (61%), and resection (80%). Median time from prostatectomy to reanastomosis was 23 months (IQR 16–42). At a median follow-up of 80 months (IQR 41–149), RFS rates were 91% at two years and 88% at five years. 30 patients (65%) required staged AUS implantation for stress urinary incontinence. PROMs, assessed in 28 patients (61%), indicated restored voiding function, absence of fecal incontinence, high treatment satisfaction, and low decision regret. Limitations include the absence of preoperative PROMs for baseline comparisons. The scoping review revealed varying success rates ranging from 60% to 92% and a significant underuse of validated PROMs.

**Conclusions:**

Transperineal reanastomosis offers an effective and lasting solution for treating recurrent or obliterative VUAS, with encouraging long-term results and favorable patient-reported outcomes, underscoring its critical role in salvage posterior urethral reconstruction after prostatectomy.

## INTRODUCTION

Despite the long-standing use of prostatectomy and radiotherapy as primary treatments for prostate cancer, these modalities continue to be associated with functional complications such as urethral stricture and vesicourethral anastomotic stenosis (VUAS) (1-3). VUAS occurs in up to 5% of patients following prostatectomy (1, 2) and is typically managed with endoscopic interventions, which generally offer acceptable short-term outcomes (4, 5). However, repeated procedures are strongly associated with an increased risk of treatment failure (6, 7). Transperineal reanastomosis serves as a salvage option for patients with refractory VUAS, particularly those with complete obliteration or those who have failed prior endoscopic management (4, 8). In up to 10% of patients, endoscopic approaches ultimately prove ineffective, making complex reconstruction necessary (9, 10). As prostate cancer therapies continue to improve and contribute to prolonged survival, the management of long-term functional complications becomes increasingly important to preserve quality of life in these patients. However, for this challenging subset of patients suffering from VUAS, the existing evidence is limited. Although quality of life is emphasized in both the European Association of Urology (EAU) guidelines for prostate cancer and urethral stricture disease (11, 12), with the latter explicitly recommending the use of patient-reported outcome measures (PROMs), most published studies are small, retrospective, and single-center in design, with short follow-up periods and minimal integration of PROMs. As a result, little is known about the long-term functional course after surgical revision, including the need for artificial urinary sphincter (AUS) implantation to manage persistent urinary incontinence. In line with current guidelines on urethral stricture disease (4, 8), which recommend reconstruction for refractory VUAS, the present study aims to fill these gaps. We describe our step-by-step technique for transperineal reanastomosis, report the longest follow-up to date for this approach, and provide a comprehensive assessment that integrates both objective outcomes and PROMs. Finally, we set our findings in the context of the existing literature by performing a scoping review on reconstruction for VUAS.

## PATIENTS AND METHODS

### Study Population and Data Extraction

This retrospective observational study was approved by the Ethics Committee of the Medical Council of Hamburg (IRB No. PV4123) and conducted in accordance with the Hamburg Hospital Act (§12.1 HmbKHG). We identified all men who underwent transperineal reanastomosis between January 2009 and December 2023. A comprehensive digital chart review was performed to collect data on demographics, etiological factors, prior VUAS treatments, previous prostate cancer therapies, prostatectomy approach, and surgical characteristics. Cross-sectional follow-up was conducted via telephone interviews and a web-based survey to gather additional outcomes.

### Study Endpoints

Study endpoints were defined as both objective and subjective outcomes. Objective outcomes included functional success, defined as retreatment-free survival (RFS) (13), with retreatment specified as any postoperative intervention for recurrent VUAS confirmed by imaging. An additional objective measure was explantation-free survival following AUS implantation. The short term (<30 days) postoperative major complication burden was also evaluated using the Clavien-Dindo classification (>grade IIIa).

Subjective outcomes were assessed through a range of validated PROMs. These included the six-item lower urinary tract symptoms (LUTS) score from the Urethral Stricture Surgery (USS) PROM (14), and the Wexner fecal incontinence score (15). Treatment satisfaction and decisional regret were further evaluated using the International Consultation on Incontinence Questionnaire–Satisfaction (ICIQ-S) (16). and the Decision Regret Scale (DRS) (17), respectively. All PROMs used in this study have undergone rigorous validation to ensure reliable representation of patient-centered outcomes. These linear scoring systems allow for comprehensive evaluation of voiding symptoms, fecal continence, as well as treatment satisfaction and decisional regret following the surgical intervention.

### Perioperative Management and Surgical Procedure

**Figure 1:**
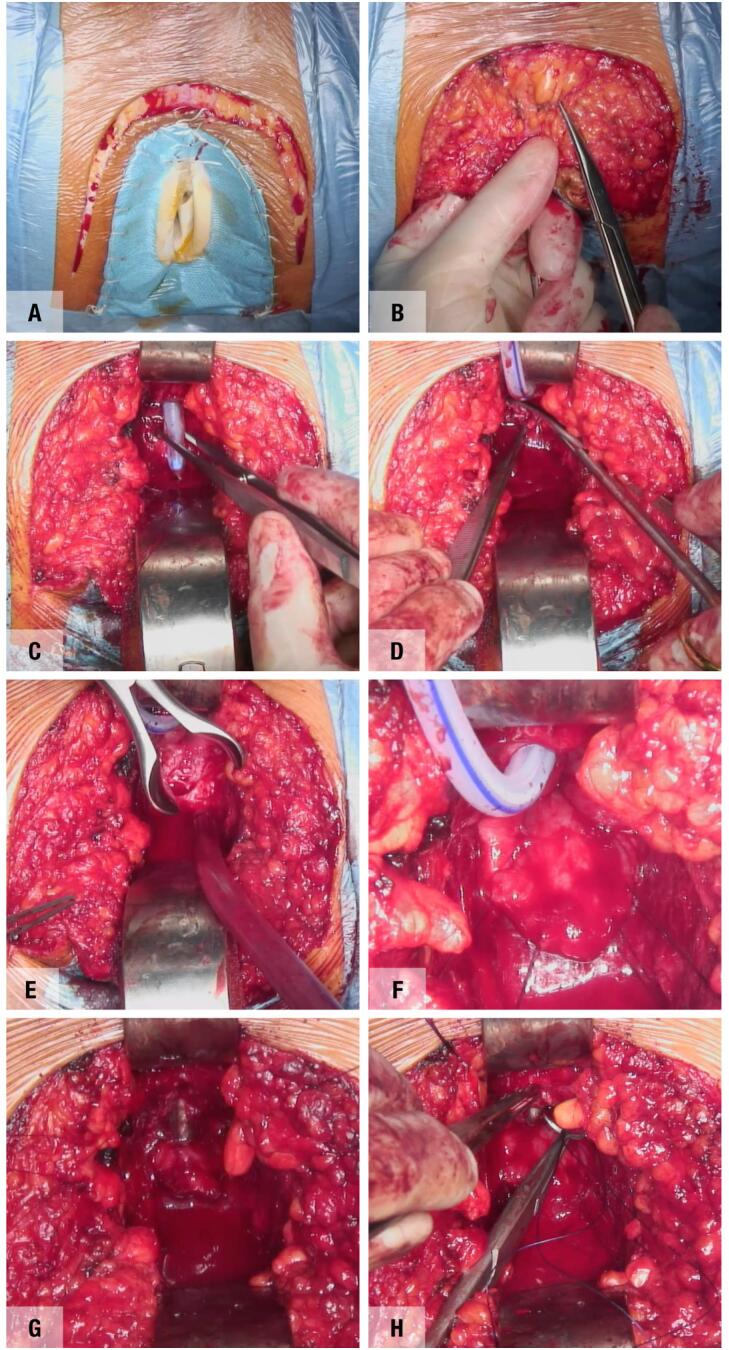
Key surgical steps of transperineal vesicourethral reanastomosis. The patient is placed in an exaggerated lithotomy position, and a half-moon perineal incision is made (1A). Dissection of the urethra is performed under digital rectal guidance (1B). After exposure of the anastomotic site and identification of the stenosis (1C), the scar tissue is meticulously excised (1D–E). The urethra and bladder are then extensively mobilized to enable a tension-free reanastomosis (1F). Eight sutures are preplaced circumferentially around the anastomotic site. The three dorsal sutures at the 11-, 12-, and 1-o'clock lithotomy positions are tied first, followed by insertion of the Foley catheter into the bladder and completion of the remaining five sutures (1G–H). The procedure concludes with leakage testing, placement of a perineal Jackson-Pratt drain, and layered closure of the wound.

Patients underwent preoperative evaluations according to our institutional protocol. This involved a detailed medical history, physical examination, urinalysis, uroflowmetry, and a combined retrograde urethrography with voiding cystourethrography, if possible. Transperineal reanastomosis is the standard salvage procedure at our institution for patients with recurrent VUAS refractory to endoscopic management or with long, obliterative stenoses unsuitable for endoscopic treatment. In accordance with our institutional practice, up to three endoscopic interventions are typically performed prior to offering open surgical reconstruction (5). In patients with a history of pelvic radiotherapy, this approach is generally avoided due to unfavorable local tissue conditions and the associated increased risk of complications and recurrence (18). In such cases, open reconstruction is considered only in highly selected, well-informed patients who explicitly wish to pursue this option despite thorough counseling and after discussion of alternative strategies, including urinary diversion procedures such as a Mitrofanoff stoma or supravesical diversion. The surgical procedure was conducted by two surgeons with extensive expertise in the field (RD, MF) as previously described (19, 20), follows the transperineal approach to the posterior urethra as described by Young (21), and is depicted step-by-step in Figure-1.

If stress urinary incontinence was present following transperineal reanastomosis, AUS implantation was performed three months postoperatively. For AUS implantation, the AMS 800™ system by Boston Scientific was used. A double-cuff configuration was placed at a location sufficiently distant from the anastomosis to maximize the urethral surface encompassed by the cuffs and thereby reducing the pressure exerted by a single cuff on a smaller urethral segment.

## Statistical Analyses

Clinical baseline characteristics were analyzed through descriptive analyses. Continuous variables are presented as medians with interquartile ranges (IQRs), while categorical variables are reported as frequencies and proportions. Median follow-up time was calculated using reverse Kaplan-Meier estimates. Kaplan-Meier curves were generated to illustrate retreatment-free survival as well as AUS explantation-free survival.

PROMs were assessed according to their respective scoring manuals. Voiding symptoms were measured using the LUTS score from the USS PROM, which ranges from 0 to 24 and includes six questions. Fecal incontinence was assessed using the Wexner fecal incontinence score, ranging from 0 to 20 and consisting of five questions. Higher scores on indicate a greater symptom burden.

Treatment satisfaction was measured with the ICIQ-S outcome score, scored from 0 to 24 across six questions addressing satisfaction with surgical outcomes, alongside a separate overall satisfaction item scored from 0 to 10. Decisional regret was assessed using a five-question questionnaire with a total score range of 0 to 100. Higher scores on the ICIQ-S indicate greater treatment satisfaction, respectively, while higher scores on the DRS indicate greater regret.

PROM scores were summarized as medians with IQRs and their distributions were visualized using violin plots.

All statistical analyses were performed using Stata® (StataCorp. 2023. Stata Statistical Software: Release 18. College Station, TX: StataCorp LLC).

Assistance in improving the English language and style was provided by ChatGPT (GPT-5, OpenAI). The tool was used exclusively for linguistic editing. No content, data interpretation, or conclusions were generated by artificial intelligence.

### Scoping Review on Outcomes After Salvage Surgical VUAS Treatment

**Figure 2:**
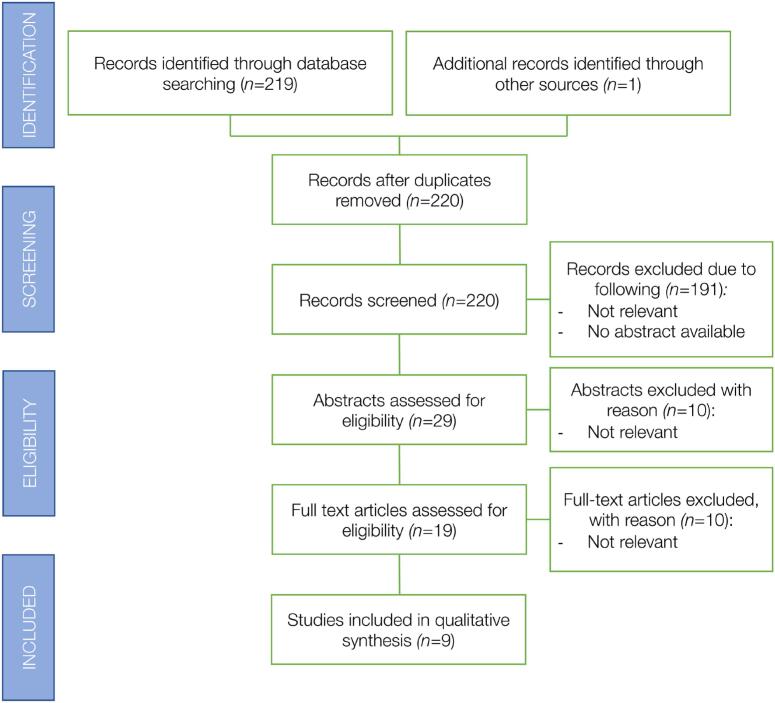
Preferred Reporting Items for Systematic Reviews and Meta-Analyses flow diagram illustrating the article selection process for the scoping review on outcomes after vesicourethral reanastomosis.

In order to provide a broader context for our findings, a comprehensive literature review was conducted in August November 30, 2024. This review involved searching the databases of PubMed with the objective of identifying studies that reported on outcomes following complex VUAS reconstruction. The search strategy employed the terms "vesicourethral anastomosis stenosis" OR "urethrovesical anastomosis stenosis" OR "prostatectomy anastomosis stenosis" OR "prostatectomy anastomotic stricture" OR "bladder neck contracture" AND "repair" OR "reconstruction" OR "revision" OR "urethroplasty". Our analysis encompassed all original articles that reported on outcomes following open, laparoscopic, or robotic VUAS repair. Additionally, we meticulously screened the reference lists of pertinent articles to identify any additional studies meeting our criteria. Only series with at least 10 patients were considered. A Preferred Reporting Items for Systematic Reviews and Meta-Analyses (PRISMA) flow diagram was created to elucidate our search strategy (Figure-2). The selected studies were tabulated, providing information on authors, publication year, cohort size, surgical approach, length of follow-up, success rate and definition, and the specific (validated) questionnaires used.

## RESULTS

### Clinical Baseline Characteristics

A total of 46 transperineal vesicourethral reanastomoses were performed at our institution between January 2009 and December 2023. The baseline characteristics of the study cohort are summarized in Table-1. The participants had a median age of 69 years (IQR 65–72) and a median body mass index (BMI) of 28 kg/m^2^ (IQR 26–32). The most prevalent preexisting medical conditions were hypertension (57%) and coronary artery disease (22%).

**Table 1:** Clinical baseline characteristics in 46 men undergoing transperineal excision and primary reanastomosis for recurrent or obliterative vesicourethral anastomotic stenosis between January 2009 and December 2023 at a tertiary reconstructive referral center.

Baseline and surgical characteristics		
Patients, n (%)		46 (100)
Age at surgery (yr), median (IQR)		69 (65–72)
BMI, median (IQR)		28 (26–32)
Comorbidities, n (%)		
	Diabetes	7 (15)
	Hypertension	26 (57)
	Coronary artery disease	10 (22)
	COPD	2 (4.4)
	Prior pelvic radiotherapy	1 (2.2)
Prostatectomy approach, n (%)		
	Open	39 (85)
	Robotic	7 (15)
Previous VUAS treatments, n (%)		
	Any prior endoscopic intervention	44 (96)
	Endoscopic dilation	8 (17)
	Transurethral incision	28 (61)
	Transurethral resection	37 (80)
	Number of previous endoscopic interventions, median (IQR)	3 (2–5)
Previous TURP, n (%)		1 (2.2)
Time from prostatectomy to reanastomosis (mo), median (IQR)		23 (16–42)
Operative time (min), median (IQR)		115 (99–132)

BMI = body mass index; COPD = chronic obstructive pulmonary disease; IQR = interquartile range; TURP = transurethral resection of the prostate; VUAS = vesicourethral anastomotic stenosis.

The majority of patients (85%) had undergone open radical prostatectomy, with the remainder having undergone robot-assisted radical prostatectomy. Nearly all patients (96%) had received prior endoscopic interventions for VUAS with a median of 3 (IQR 2–5) per patient. Two patients (4.3%) did not undergo prior endoscopic treatment, as they presented with long, highly obliterative VUAS not amenable to endoscopic management. The most commonly performed procedures included transurethral resection (80%), transurethral incision (61%), and endoscopic dilation (17%).

The median time from prostatectomy to first VUAS intervention was seven months (IQR 4-31) and the median time from radical prostatectomy to transperineal reanastomosis was 23 months (IQR 16–42), median operative time was 115 minutes (IQR 99–132). There were no major short term postoperative complications > grade IIIa according to the Clavien-Dindo classification.

### Retreatment-free Survival and Staged AUS Implantation

**Figure 3:**
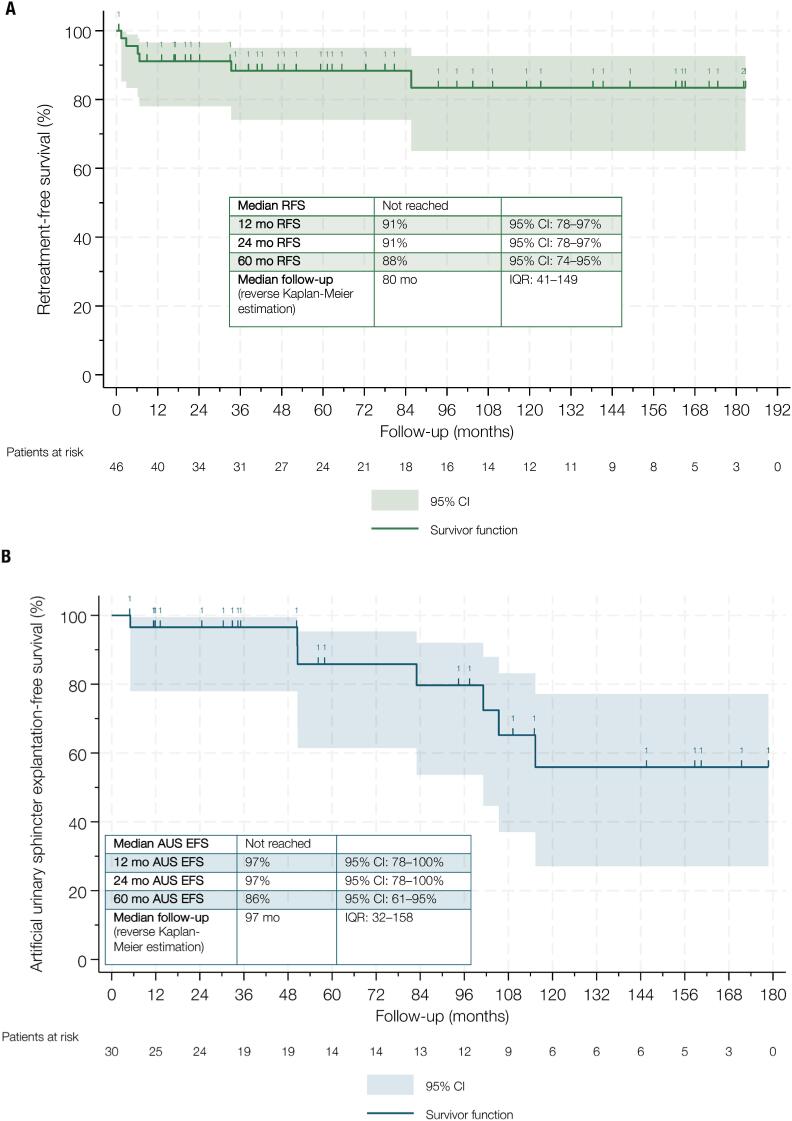
Kaplan-Meier curves depicting (A) retreatment-free survival in 46 patients undergoing transperineal reanastomosis for recurrent or obliterative vesicourethral anastomotic stenosis and (B) explantation-free survival in 30 patients undergoing AUS implantation following transperineal reanastomosis. AUS, artificial urinary sphincter; CI, confidence interval; EFS, explantation-free survival; IQR, interquartile range; RFS, retreatment-free survival.

At a median follow-up of 80 months (IQR 41–149), six patients (13%) required retreatment, resulting in RFS rates of 91% at two years and 88% at five years (Figure-3A). Of these, five patients underwent additional endoscopic interventions (transurethral resection or incision), while one patient required bladder neck closure followed by suprapubic catheter diversion.

ICIQ = International Consultation on Incontinence Questionnaire; IQR = interquartile range; USS PROM = Urethral Stricture Surgery Patient-Reported Outcome Measure.

Additionally, 30 patients (65%) received staged artificial urinary sphincter (AUS) implantation to address stress urinary incontinence. Of the 16 patients without AUS implantation at last follow-up, one (6.3%) had died, four were lost to follow-up (25%), four had low-capacity bladders (25%), two had significant detrusor overactivity (13%), one experienced recurrent VUAS (6.3%), and four (25%) were scheduled for AUS implantation that had not yet been performed. At a median follow-up of 97 months (IQR 32–158), seven AUS explantations (23%) were necessary. The reasons for explantation included five cases of urethral erosion, one case of relative urethral stenosis, and one case with an unknown cause managed at an external hospital. This corresponds to estimated AUS explantation-free survival rates of 97% at two years and 86% at five years (Figure-3B).

## PROMs

PROMs were assessed at a median of 78 months (IQR 41–138) following surgery in 28 patients, representing 61% of the cohort. It is noteworthy that five patients (11%) had passed away during the follow-up period.

**Figure 4:**
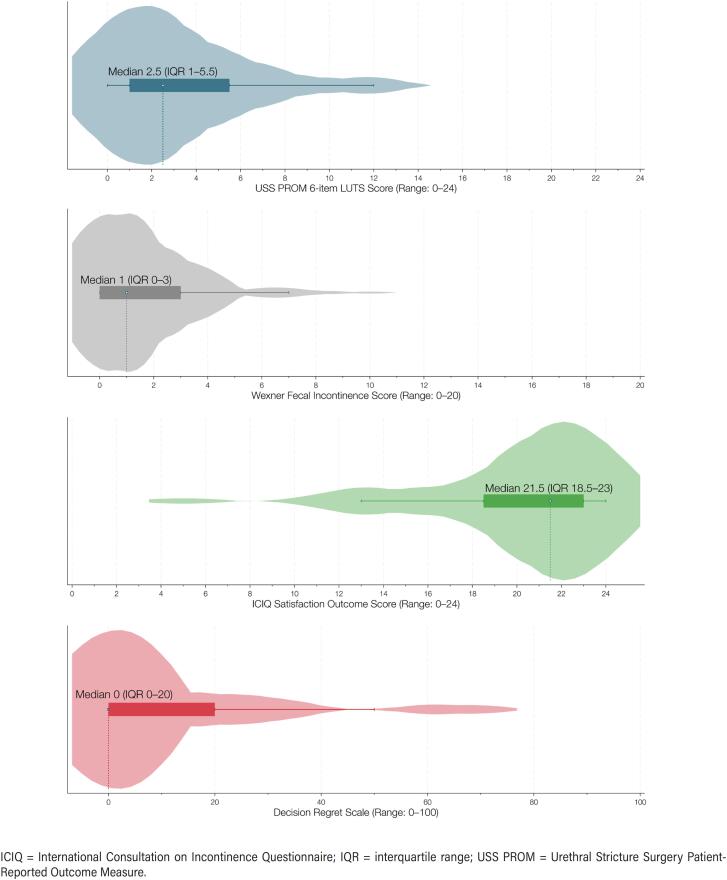
Violin plots illustrating the distribution of scores for validated patient-reported outcome measures in 28 of 46 patients undergoing transperineal vesicourethral reanastomosis.

The distribution of all evaluated PROMs is illustrated in Figure-4. The median (IQR) scores for each PROM were as follows: the USS PROM six-item LUTS score (reference range: 0–24) was 2.5 (1–5.5), reflecting a low burden of voiding symptoms; the Wexner fecal incontinence score (reference range: 0–20) was 1 (0–3), indicating minimal fecal incontinence; the ICIQ-S outcome score (reference range: 0–24) was 21.5 (18.5–23), suggesting high satisfaction with surgical outcomes; the ICIQ-S satisfaction with surgery item (reference range: 0–10) was 9 (7.5–10), further highlighting positive treatment satisfaction; and the DRS (reference range: 0–100) was 0 (0–20), demonstrating low levels of regret associated with the treatment decision.

### Scoping Review on Outcomes After Salvage Surgical VUAS Treatment

Following the selection process, nine articles comprising 189 patients that met our inclusion criteria were identified (20, 22-29). The reported success rates, predominantly defined as reintervention-free survival, ranged from 60% to 92%, with median/mean follow-up durations varying between 10 and 76 months (Table-2).

**Table 2:** Publications on outcomes after complex VUAS reconstruction as identified by a scoping literature review on November 30, 2024.

First author	Year of publication	No. of patients	Surgical approach	Median/Mean follow-up (mo)	Success rate (%)*	PROMs
Pfalzgraf et al. (28)	2012	20	Open retropubic reanastomosis	59	60	NR
Simonato et al. (29)	2012	11	Open Solovov-Badenoch 'pull-through urethroplasty'	65	91	NR
Nikolavsky et al. (27)	2014	12	Open abdominal reanastomosis (7), open transperineal reanastomosis (3), open abdominoperineal reanastomosis (2)	76	92	NR
Schuettfort et al. (20)	2017	23	Open transperineal reanastomosis	45	87	I-PSS and QOL item, ICIQ-UI SF, EQ-5D-3L
Giúdice et al. (26)	2019	20	Open transperineal reanastomosis (10), open transabdominal reanastomosis (10)	10	70	NR
Şimşek et al. (25)	2021	16	Open transperineal reanastomosis (12), robotic perineoscopic reanastomosis (4)	13	81	NR
Bearrick et al. (24)	2022	10	Open primary scar excision and re-anastomosis (5), YV plasty (3), downward rotational bladder advancement (2)	24	90	NR
Shakir (23)	2022	32	Robotic retropubic reanastomosis	12	75	NR
Sterling et al. (22)	2024	45	Open dorsal onlay buccal mucosal graft urethroplasty	21	84	I-PSS and QOL item, Global Response Assessment

* Defined as reintervention-free survival; referred to as patency in Nikolavsky et al. (2014) and Giúdice et al. (2019). ICIQ-UI SF = International Consultation on Incontinence Questionnaire-Urinary Incontinence Short Form; I-PSS = International Prostate Symptom Score

Apart from our earlier preliminary series on transperineal reanastomosis (20), only one study incorporated validated PROMs in their analysis, including the International Prostate Symptom Score (I-PSS) with the QOL item, and the Global Response Assessment to evaluate patient satisfaction (22). However, differences in follow-up durations, endpoint definitions, and cohort heterogeneity limit the comparability of these outcomes across studies.

## DISCUSSION

Refractory VUAS is a challenging condition that significantly affects quality of life in prostate cancer survivors and often necessitates complex surgical reconstruction to avoid urinary diversion or long-term suprapubic catheterization. This series presents the largest single-center cohort with the longest follow-up to date for the treatment of complex post-prostatectomy VUAS following failed endoscopic management or complete stenotic obliteration. By employing a predominantly staged approach—transperineal vesicourethral reanastomosis followed by AUS implantation—we achieved effective restoration of voiding function, high patient satisfaction, and negligible decision regret.

Our retreatment-free survival rates align with the >80% success reported in two-thirds of the existing literature on complex VUAS reconstruction. Two factors likely contributed to this success: complete excision of scar tissue, which restores an uncompromised surgical field for optimal reconstruction (28), and the absence of prior pelvic radiation in nearly all patients, as radiotherapy negatively impacts surgical outcomes. For VUAS involving the urinary sphincter, a transperineal approach is often preferred, as it provides optimal anatomical exposure of critical structures. In contrast, transabdominal or robot-assisted approaches may be more suitable when the VUAS extends toward the bladder with minimal or no sphincter involvement (30). The choice of surgical approach should be guided by the location and extent of the VUAS, both of which are influenced by the degree of membranous urethra preservation during prostatectomy and the number of prior endoscopic interventions. Moreover, variations in vesicourethral anastomosis technique may further impact scarring into the membranous urethra, as different approaches appear to carry varying risks for VUAS development (1). In our department, up to three prior endoscopic treatments are typically performed. As a result, VUAS in our cohort often exhibit significant scar tissue and frequently extend into the membranous urethra, precluding sphincter preservation in many cases and limiting the feasibility of a transabdominal approach, as a transperineal incision becomes necessary due to the distal extension of the stenosis. To improve patient selection in the future, a classification system for VUAS based on anatomical location and prior treatments would be highly valuable.

The AUS explantation-free survival rate in our cohort is slightly higher than those reported in a recent large French cohort (31). This difference may be partly explained by the lower incidence of prior pelvic radiotherapy in our cohort, as radiotherapy is known to increase reintervention rates. While no systematic review has reported reintervention-free survival for AUS implantations, a recent systematic review highlighted the significant concern of reinterventions following AUS implantation by reporting pooled reoperation rates of 22% in the short term (6–36 months) and 36% in the long term (>36 months), with a mean follow-up of 19 months (32).

According to our scoping review, our series is the first to assess patient-centered outcomes for anastomotic repair of refractory VUAS. Several validated PROMs were utilized, covering key functional domains such as voiding and fecal continence, as well as broader outcome measures, including treatment satisfaction and decision regret. Notably, the postoperative LUTS symptom burden observed in our cohort is comparable to that reported for patients undergoing anterior urethroplasty in the pivotal USS PROM study (14). Our excellent results in fecal continence, as measured by the Wexner incontinence score, which has been shown to be associated with quality of life, were anticipated but remain significant, as pelvic surgeries and transperineal approaches can impact fecal continence (33).

Arguably of greater impact than PROMs for functional outcomes are overall patient satisfaction and decision regret—aspects that have been largely unexplored in the context of refractory VUAS. This is especially crucial when multiple treatment alternatives are available, requiring patients to make informed choices among them. The relatively novel and validated ICIQ-S effectively captures a broad range of critical perioperative aspects by aggregating patient feedback into a comprehensive overall score (16). It evaluates not only the surgical outcome through questions like "How would you rate the outcome of your surgery?" but also explores the patient's emotional well-being post-surgery, their ability to resume normal life, and their willingness to undergo the procedure again or recommend it to others. In combination with the DRS, which evaluates decision regret following a patient's choice through five questions addressing whether the decision was deemed "right", "regretted", "worth repeating", "harmful", or "wise," these two PROMs provide a comprehensive overview of how patients assess their treatment. Despite the demanding nature of a staged procedure requiring two invasive surgeries, treatment satisfaction in our cohort remains high, with minimal decision regret, highlighting the fundamental rationale for this therapeutic approach.

Our study has several limitations. Its retrospective design precluded assessment of urinary continence after prostatectomy but before the first endoscopic intervention, which would have allowed evaluation of changes in stress urinary incontinence over the course of endoscopic therapies and transperineal reanastomosis. The relatively small sample size limited the use of multivariable regression analyses. Additional limitations include the cross-sectional nature of follow-up, the absence of preoperative PROMs for baseline comparisons, and potential recall bias. Although the PROM response rate of 61% is comparable to similar long-term retrospective studies, the possibility of response bias should be acknowledged. Nevertheless, we believe this series makes a significant contribution to the literature by demonstrating highly satisfactory outcomes for open transperineal vesicourethral reanastomosis in a patient cohort facing a complex refractory post prostatectomy late complication with likely long-term survival, highlighting that definitive urinary diversion or long-term suprapubic catheterization should not be considered the ultimate solutions for these patients.

## CONCLUSIONS

Open transperineal reanastomosis is a valuable and durable option for managing recurrent or obliterative post-prostatectomy VUAS refractory to endoscopic therapy, demonstrating favorable long-term outcomes and positive patient-reported experiences. This largest series to date underscores the procedure's critical role in salvage posterior urethral reconstruction for long-term prostate cancer survivors.

## Data Availability

All data generated or analysed during this study are included in this published article
